# A Community Health Worker Day in Rural Chiapas, Mexico, in a Nutshell

**DOI:** 10.4269/ajtmh.23-0727

**Published:** 2024-04-09

**Authors:** Zeus Aranda

**Affiliations:** Partners In Health Mexico/Compañeros En Salud, Chiapas, México

My phone alarm goes off, 4:30 am. I quickly put on a T-shirt and shorts and head excitedly to Bere’s house. Today is my first day following Bere, a community health worker, as part of a time and motion study at Partners In Health, Mexico. To my surprise, it’s cold. I regret not bringing a sweater. The flashlight on my phone cuts through utter darkness, barely illuminating the road. As I walk past, dogs bark, disconcerted by the unexpected footsteps of a stranger, interrupting the silence of the early morning. As their howls die down, the trickling of the stream next to the road keeps me company in the silence. After half an hour of walking downhill, I arrive at Bere’s house, made of brick and painted in lemon yellow. Bere waits for me at the entrance, well bundled up and wearing a hat to face the cold, much better prepared than I am. She invites me into her house and offers me a delicious coffee harvested by her father. After clearing our heads, we leave the house for the first home visit of the day.

We walk for about 30 minutes in the dark, traveling along a dirt road full of potholes and surrounded by jungle vegetation. To reach the house, we have to climb a small hill, which Bere overcomes with great agility while I lag a few steps behind. It is a small house made of wood and corrugated iron, surrounded by paint buckets used as flowerpots. Bere knocks on the door. Soon, an elderly, petite woman opens the door with a broad smile that accentuates the wrinkles on her face. The woman, called Doña Eulalia, pulls up some chairs for us to sit on and brings us a cup of coffee. While Bere prepares the material to take Doña Eulalia’s glucose, the woman explains the economic problems her family is facing. The lack of rain this year has affected the coffee harvest, and the increase in the price of agricultural inputs has left them with very little income. It will be difficult for her, her husband, and their two young grandchildren to cover their basic needs. Bere finishes with the capillary glucose reading, which indicates higher than healthy values. Bere advises Doña Eulalia to avoid bread and sugary drinks. Given the family’s difficult economic situation, Bere decides to inquire about Doña Eulalia’s psychoemotional state. She expresses difficulty falling asleep, apathy, and listlessness. Then, Bere, with a serious and concentrated look on her face, follows the instructions on her tablet to screen her for anxiety and depression. Entering Doña Eulalia’s answers into the tablet, the software indicates that the patient has symptoms for both conditions, which Bere sensitively communicates to Doña Eulalia. Bere schedules a consultation for her at the clinic and recommends that she spend time doing whatever activities she finds most pleasurable, such as playing with her grandchildren, tending to her plants, or knitting. Doña Eulalia nods her head and looks into Bere’s eyes saying “thank you for everything.” I feel conflicted, privileged to be able to witness this endearing moment but also guilty of breaking their intimacy. We both say goodbye to Doña Eulalia, who bids us farewell with the smile that greeted us.

We return to Bere’s hamlet. The sun begins to rise timidly through the mountains, as the morning mist dissipates. The roosters welcome us with their crowing as we watch the children leave their homes to go to school. Now we head to the second visit of the day. On the outskirts of the hamlet, crossing a river, we arrive at a small adobe house. Bere calls the man of the house, Don Alberto, but he does not answer. It looks like there is no one home, the windows and doors are closed, and we hear no noise other than the current of the river. Bere knocks on the door and it opens without resistance. We find Don Alberto, sitting in a chair facing the wall, eating a piece of fruit. Don Alberto does not notice our presence until Bere touches his shoulder, which makes him jump. They embrace while Don Alberto gets a smile on his face. This older gentleman lives alone. He has suffered for years from hypertension, has difficulty hearing, and has cataracts that prevent him from seeing. Bere starts the visit with a routine hypertension follow-up, including blood pressure measurement and dispensing of medication. Then, Bere asks the gentleman, shouting, if he has any news about possible cataract surgery. Don Alberto replies “no”; the doctor at the health center will let him know when a surgical campaign comes to the nearest hospital, about 4 hours away. Even then, Don Alberto will need to find a way to get there and cover the cost of the surgery. Bere shakes her head in denial while whispering to me that it has been about a year since Don Alberto lost his vision almost completely and he still has not been able to have the surgery. A feeling of great helplessness invades me when I think how easily in a different context Don Alberto could enjoy perfect vision. Bere tells Don Alberto that Partners In Health can help him cover the cost of the intervention. Before the end of the visit, Bere asks Don Alberto about his children, who migrated to the United States four years ago. Don Alberto does not know when they will return. He misses them very much. Thanks to their support, he can make ends meet, since he has been unable to work for the past year because of his vision problems. Bere carefully places some cereal bars and fruit in Don Alberto’s hands, and we say goodbye to him.

We head out for our third visit of the day. We arrive at a house in the center of the hamlet; it is a large house, made of masonry, painted in an ochre color. A neighbor tells us that Doña Chayo has gone out to run an errand and will be back soon. After 40 minutes of waiting in the sun and soaking my T-shirt in sweat, she arrives. She invites us into her living room, a large, dimly lit room with high ceilings and walls decorated with graduation photographs. At one end of the room, I catch a glimpse of a young girl, with a lost look, immobile in a wheelchair. Doña Chayo tells us that she sees her daughter looking sadder and duller than usual, that her epilepsy medication has recently been changed, and that this has affected her. Since her daughter has been like this, Doña Chayo has had to carry all her weight to put her to bed or go to the bathroom. As a result of this situation, she has been suffering from severe back pain for a couple of months now, in addition to having relapsed into depression. Bere makes a worried face as she thinks silently for a few moments. She decides to give her some painkillers and recommends going for walks and keeping busy with activities that make her feel good. She also reminds her of the importance of making her depression follow-up visits at the clinic. Doña Chayo tells us that her daughter’s wheelchair has been failing. A year and a half ago, she requested a new chair from the social assistance office. She was told that they would get it in a month, but they still have no news. With this information, Bere and I look at each other with discouragement; there is little Bere can do to get a new chair. Bere encourages Doña Chayo to keep trying and tells her that she will stop by the office to reinforce the request. Doña Chayo thanks Bere for her support and gives us some beautiful mangoes as we leave.

We arrive at our last stop, a shack made of wood and corrugated iron on the outskirts of the hamlet, down a hillside next to a makeshift dunghill. A woman is cooking over a campfire, which is emitting all the smoke towards the entrance of the house. Next to her are three children. After welcoming us and offering us a seat, Nayelli apologizes for the unhygienic conditions of the place. Running water had not reached the area in four days, and she had not been able to shower her children, wash their clothes, or clean the house. The little water they have is used for cooking and drinking. Nayelli is worried about one of her three children. At five years old, he is not yet able to speak, but he is very skilled and learns very easily. Bere tells her that it may be related to hearing problems and schedules an appointment for him at the clinic. Then, Bere performs a test to identify malnutrition in the three children, according to which all three appear to be undernourished. Nayelli tells us that they do not have enough resources to buy nutritious food. According to Nayelli, her husband—who can be heard snoring inside the house—drinks a lot and spends much of his salary on alcohol. Nayelli bursts into tears. Bere, determined, approaches her and embraces her. I feel anger, helplessness, sadness, and a compelling need to do something paired with heavy feelings of discouragement at the enormity of the obstacles Nayelli is experiencing. Bere hands Nayelli several cereal bars, oral serum, and several pieces of fruit and tells her she will bring her more the next day. With that we say goodbye to Nayelli and the children and return to Bere’s house.

As we walk, I think about all that I have witnessed this day. It is touching to see the work of accompaniment carried out by community health workers, which transcends the health needs of the population. On the other hand, I cannot imagine the frustration of community health workers as they face the daily difficulties of their families and neighbors, which cannot be solved with “a pill” but are a consequence of the global economy, climate change, corrupt and ineffective public institutions, and the marginalization of certain social sectors by the system. When I ask Bere how she deals with this situation, she tells me “We know we can’t eliminate all of people’s problems, but it gives me satisfaction to go to sleep thinking that I have helped to alleviate even a little bit of suffering within my community.”

